# Cardiovascular benefits of SGLT2 inhibitors in type 2 diabetes, interaction with metformin and role of erythrocytosis: a self-controlled case series study

**DOI:** 10.1186/s12933-022-01520-w

**Published:** 2022-06-03

**Authors:** Carlos King Ho Wong, Kristy Tsz Kwan Lau, Eric Ho Man Tang, Chi Ho Lee, Carmen Yu Yan Lee, Yu Cho Woo, Ivan Chi Ho Au, Kathryn Choon Beng Tan, David Tak Wai Lui

**Affiliations:** 1 Department of Family Medicine and Primary Care, School of Clinical Medicine, Li Ka Shing Faculty of Medicine, The University of Hong Kong, Hong Kong, SAR China; 2 Department of Pharmacology and Pharmacy, Li Ka Shing Faculty of Medicine, The University of Hong Kong, Hong Kong, SAR China; 3 Laboratory of Data Discovery for Health Limited (D24H), Hong Kong Science Park, New Territories, Hong Kong, SAR China; 4 Department of Medicine, School of Clinical Medicine, Li Ka Shing Faculty of Medicine, The University of Hong Kong, Hong Kong, SAR China

**Keywords:** Cardiovascular disease, Erythrocytosis, Heart failure, Metformin, Self-controlled case series, Sodium-glucose cotransporter-2 inhibitor, Type 2 diabetes

## Abstract

**Background:**

Sodium-glucose cotransporter-2 inhibitors (SGLT2i) have proven cardiovascular benefits in patients with type 2 diabetes (T2D). This self-controlled case series study aims to evaluate whether metformin use and SGLT2i-associated erythrocytosis influence its cardiovascular benefits.

**Methods:**

T2D patients with metformin and/or SGLT2i prescriptions between 2015 and 2020 were identified from the Hong Kong population. Study outcomes were composite cardiovascular diseases (CVD), coronary heart disease (CHD), hospitalisation for heart failure (HHF), stroke, and erythrocytosis. Risk periods were patient-time divided into four mutually exclusive windows: (i) ‘baseline period’ of metformin use without SGLT2i; (ii) pre-SGLT2i period; (iii) exposure to SGLT2i without metformin; and (iv) exposure to the drug combination. Another SCCS model was applied to evaluate the association between erythrocytosis and cardiovascular outcomes regarding SGLT2i exposure. Four mutually exclusive risk periods included (i) SGLT2i exposure with erythrocytosis; (ii) SGLT2i exposure without erythrocytosis; (iii) absence of SGLT2i exposure with erythrocytosis; and (iv) absence of SGLT2i exposure without erythrocytosis. Incidence rate ratios (IRR) of events at different risk periods were estimated using conditional Poisson regression model.

**Results:**

Among 20,861 patients with metformin and/or SGLT2i prescriptions, 2575 and 1700 patients with events of composite CVD and erythrocytosis were identified, respectively. Compared to metformin use without SGLT2i, SGLT2i initiation was associated with lower risks of composite CVD, CHD, and HHF—regardless of the presence (CVD: IRR = 0.43, 95% CI 0.37–0.51; CHD: IRR = 0.44, 95% CI 0.37–0.53; HHF: IRR = 0.29, 95% CI 0.22–0.40; all p < 0.001) and absence of concomitant metformin (CVD: IRR = 0.31, 95% CI 0.20–0.48; CHD: IRR = 0.38, 95% CI 0.25–0.59; HHF: IRR = 0.17, 95% CI 0.09–0.31; all p < 0.001); while SGLT2i was neutral on stroke risk. Compared to metformin-SGLT2i combination, exposure to SGLT2i alone was associated with comparable risks of all cardiovascular outcomes (all p > 0.05). Incidence rates of erythrocytosis at baseline, SGLT2i without and with metformin use periods were 0.75, 3.06 and 3.27 per 100 person-years, respectively. SGLT2i users who developed erythrocytosis had lower risk of HHF (IRR = 0.38, 95% CI 0.14–0.99, p = 0.049) than those who did not.

**Conclusions:**

Our real-world data suggested that SGLT2i-associated cardiovascular benefits were not attenuated by metformin use. Further studies will delineate the role of erythrocytosis as a surrogate marker of SGLT2i-associated cardiovascular benefit in reducing HHF.

**Supplementary Information:**

The online version contains supplementary material available at 10.1186/s12933-022-01520-w.

## Introduction

Cardiovascular diseases (CVD) are major causes of morbidity and mortality in patients with type 2 diabetes (T2D) [1]. As sodium-glucose cotransporter-2 inhibitors (SGLT2i) demonstrate significant cardiovascular benefits in T2D patients across multiple cardiovascular outcome trials [2–7], current guidelines including the latest one by the American Diabetes Association (ADA) recommend the use of SGLT2i in T2D patients with established or at high risk of CVD independent of glycaemic control, regardless of baseline metformin use [8–11]. In particular, the European Society of Cardiology has even suggested the upfront use of SGLT2i monotherapy in T2D patients with established or at high risk of CVD [9]. Multiple postulated mechanisms underlie the cardiovascular benefits of SGLT2i, including augmented natriuresis and osmotic diuresis, restoring tubuloglomerular feedback and reducing glomerular hyperfiltration, limiting renin–angiotensin–aldosterone system (RAAS) activation, activating sirtuin-1 (SIRT-1) and hypoxia-inducible factors (HIF), improving myocardial energetics and remodelling, as well as reducing inflammation and fibrosis, all independent of improved glycaemic control [2, 12–14]. Interestingly, mediation analyses have recently identified increases in haematocrit, haemoglobin or erythrocyte concentration as the most important mediators of SGLT2i in reducing the risks of heart failure (HF) and cardiovascular death among T2D patients [15, 16]. Nonetheless, SGLT2i-associated erythrocytosis has been increasingly recognised and reported in case series [17]. As evidence has suggested an association between erythrocytosis and increased major adverse cardiovascular events (MACE) [18], it is clinically relevant to delineate the extent of SGLT2i-associated erythrocytosis in a population-based cohort, and to examine whether SGLT2i users who develop erythrocytosis may have different cardiovascular risk profiles. These results will provide valuable information to diabetes care providers.

Metformin is generally considered the first-line therapy based on its safety, efficacy, availability and affordability [8]. There are recent concerns regarding the possibility of metformin in attenuating SGLT2i-associated benefits on cardiovascular death or hospitalisation for HF (HHF), observed from the attenuation in cardiovascular benefits among canagliflozin users by baseline metformin therapy in the CANVAS Program (p for interaction = 0.03) [19] and possibly among empagliflozin users by baseline metformin therapy in a prespecified analysis of the EMPA-REG OUTCOME trial (p for interaction = 0.07) [20]. On the other hand, a post-hoc analysis of the DECLARE-TIMI 58 trial suggested that the cardiovascular benefits associated with dapagliflozin was consistently observed in T2D patients with or without baseline metformin therapy [21]. Mechanistically, the partial overlap of action between metformin and SGLT2i may explain this potential attenuation, both being able to activate AMP-activated protein kinase (AMPK) in the myocardium [22, 23].

Hence, we set out this self-controlled case series (SCCS) study that aims to evaluate, using real-world data of patients with T2D, the cardiovascular benefits of SGLT2i in terms of (i) the potential interaction with metformin use, and (ii) the role of erythrocytosis.

## Methods

### Data source and study population

A territory-wide cohort of patients diagnosed with T2D and managed under the Hong Kong Hospital Authority was analysed for the period from 1st January 2015 to 31st December 2020. Electronic medical records of patients were extracted from the Hong Kong Hospital Authority, a statutory body that provides public healthcare services and manages all public hospitals and their ambulatory clinics in Hong Kong.

### Exposure and study outcomes

Patients who had received SGLT2i and/or metformin after the diagnosis of T2D were included in the current analysis. Patients with T2D were identified by International Classification of Primary Care, Version 2 (ICPC-2) code of T90 or International Statistical Classification of Diseases and Related Health Problems, 9th Revision, Clinical Modification (ICD-9-CM) codes of 250.×0 or 250.×2. Patients with stage 4–5 chronic kidney disease, dialysis, renal transplant, or erythrocytosis before the start of observation (1st January 2015) were excluded.

Study outcomes were composite CVD, coronary heart disease (CHD), HHF, stroke, and erythrocytosis. Patients with CVD were defined as a composite outcome of CHD, HHF, stroke, and cardiovascular death. These were identified by corresponding diagnostic codes from ICD-9-CM, International Statistical Classification of Diseases and Related Health Problems, 10th Revision, Clinical Modification (ICD-10-CM), and ICPC-2 (Additional file 1: Table S1). Erythrocytosis was defined as (i) haemoglobin > 16.5 g/dL or haematocrit > 49% for men; or (ii) haemoglobin > 16.0 g/dL or haematocrit > 48% for women [24].

### SCCS study design

This study was conducted with the SCCS method. This approach was applied to investigate the association of single use of SGLT2i or metformin, or combination use of the drugs for T2D patients and the risks of study outcomes. The SCCS study design relies on the comparisons within individuals who have experienced both the outcome and exposure of interest, with the participants serving as their own control [25]. Incidence rate ratios (IRR) were derived by comparing the rate of events during periods of medication exposure with the rate during all other observed time periods. The major advantage of this study design is the ability to control for the fixed confounders and time-invariant confounding that possibly vary between individuals, such as socioeconomic factors and genetic factors [26]. Besides, the SCCS approach could avoid immortal time bias by analysing the risk using Poisson-type regression with clear classification of all person-time in different exposure period [27, 28].

### Study assumptions

There were three essential assumptions in the SCCS design such that the study would be able to provide valid and unbiased estimates [26]. First, recurrent events of study outcomes among SGLT2i and metformin users were assumed to be independent. In the case that the events were dependent, the first event would be possible to increase the risk of a future event. Therefore, only the first incident event was included in this study. Second, the occurrence of an event must not alter the probability of subsequent exposure. To resolve this assumption violation, this study included a pre-exposure period (i.e. pre-SGLT2i period) so that the events would not temporarily alter the probability of drug initiation. Third, there must be no censoring by the outcome of interest. If the SCCS analyses were permissible to censor exposure by the outcomes, it could produce bias and lead to an unpredictable direction because of the violation to another SCCS design assumption and event-dependent exposure history. The study outcomes would then be affected, for instance, a higher incidence rate would be estimated while a risk period was censored by the patient’s death, and the estimated IRR would be biased downwards or upwards depending on the death occurrence in different risk periods. This would lead to an event-dependent situation and violate the assumption of SCCS study design, where an extended version of SCCS is required to adjust for censoring by applying a weighting according to the duration from an event to the end of observation.

### Exposure and risk periods

**Fig. 1:**
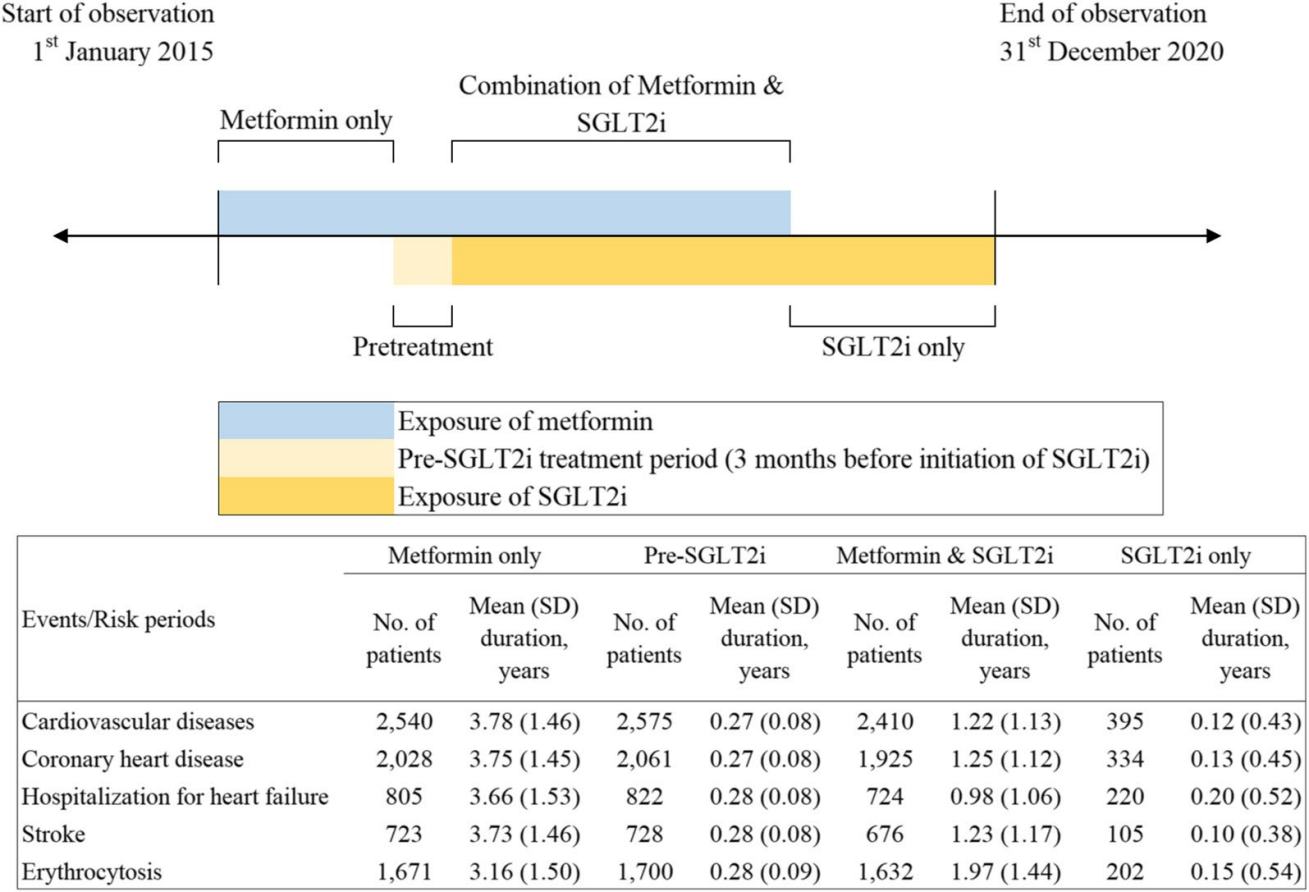
Illustration of the self-controlled case series model by metformin and SGLT2i use. The risk periods were patient time divided into four mutually exclusive windows: (i) baseline period which covered the time of metformin use without SGLT2i; (ii) pre-SGLT2i period as the pre-exposure period, which was defined as three months before each initiation of SGLT2i; exposure periods including (iii) SGLT2i use alone period; and (iv) combined use of metformin and SGLT2i period.

Patients were observed until the end of the observation period (i.e. 31^st^ December 2020). The risk periods were patient time divided into four mutually exclusive windows: (i) baseline period which covered the time of metformin use without SGLT2i; (ii) pre-SGLT2i period as the pre-exposure period, which was defined as three months before each initiation of SGLT2i; exposure periods including (iii) SGLT2i use alone period; and (iv) combined use of metformin and SGLT2i period. The pre-SGLT2i period was designed to evaluate any increased incidences of outcome events before the initiation of SGLT2i. Inclusion of the pre-SGLT2i period would attenuate the issue when events temporarily increased or decreased the probability of SGLT2i initiation, notably cardiovascular events which form the indications of its use. A pictorial representation of this SCCS approach is shown in Fig. 1.

**Fig. 2:**
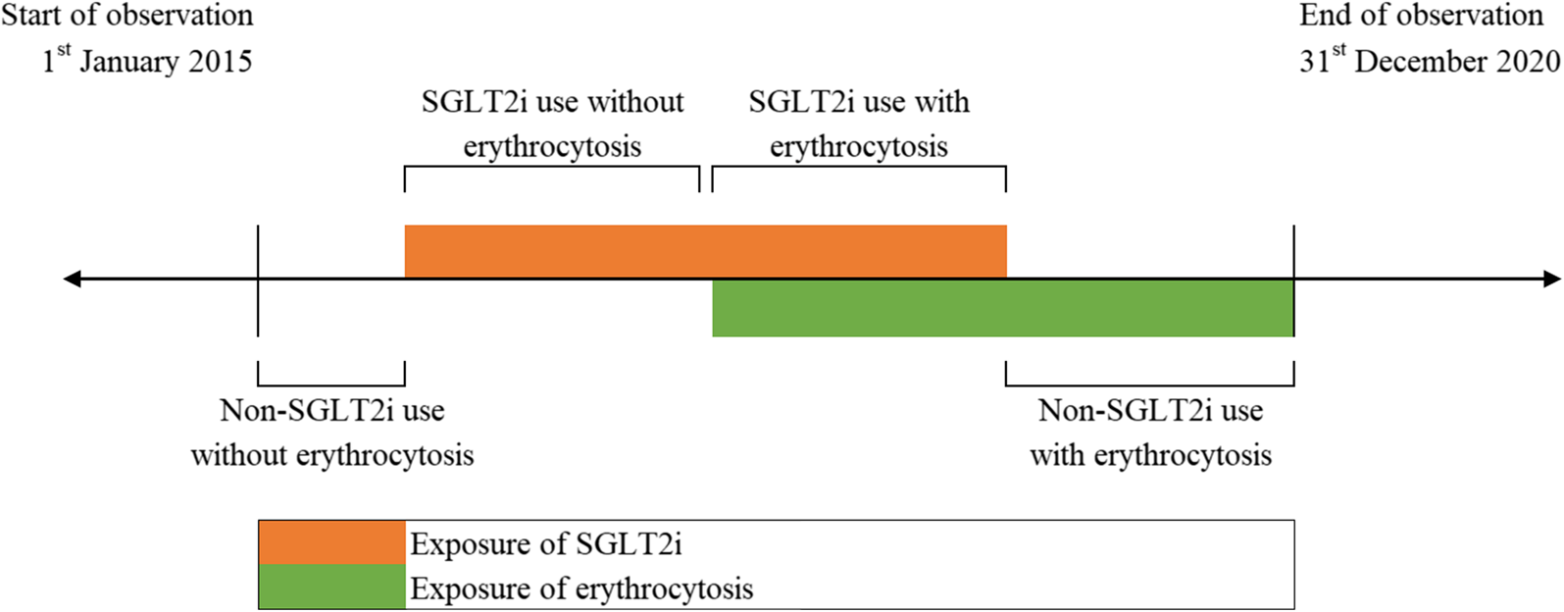
Illustration of the self-controlled case series model by exposure to erythrocytosis and SGLT2i. This model was applied to evaluate the association between erythrocytosis and cardiovascular outcomes, in the presence or absence of SGLT2i exposure. Four mutually exclusive risk periods included (i) SGLT2i exposure with erythrocytosis; (ii) SGLT2i exposure without erythrocytosis; (iii) absence of SGLT2i exposure with erythrocytosis; and (iv) absence of SGLT2i exposure without erythrocytosis

Another SCCS model was applied to evaluate the association between erythrocytosis and cardiovascular outcomes, in the presence or absence of SGLT2i exposure. Four mutually exclusive risk periods included (i) SGLT2i exposure with erythrocytosis; (ii) SGLT2i exposure without erythrocytosis; (iii) absence of SGLT2i exposure with erythrocytosis; and (iv) absence of SGLT2i exposure without erythrocytosis. The pictorial representation of this model is illustrated in Fig. 2.

### Statistical analyses

Descriptive statistics, namely proportion (%) and mean ± standard deviation, were used to summarise the characteristics of patients who had incident outcome events during the observation period. Incidence rates (IR), in terms of number of events per 100 person-years, of composite CVD, CHD, HHF, stroke, and erythrocytosis over different treatment periods were calculated. IRR and their 95% confidence intervals (CI) of events for different risk periods compared with the baseline period were estimated using conditional Poisson regression model with an offset for the length of the risk period, with adjustment of time-varying factors (namely age and the use of other anti-diabetic agents [sulfonylureas, thiazolidinediones, dipeptidyl peptidase-4 inhibitors, GLP1 receptor agonists, and insulin]).

Two subgroup analyses were performed: (i) to analyse any differential effects of two distinct SGLT2i, dapagliflozin and empagliflozin, on the risks of cardiovascular outcomes and erythrocytosis (subgroup analysis on canagliflozin was precluded due to its limited use in the current study); and (ii) to evaluate the impact of adding SGLT2i to metformin users when glycated haemoglobin (HbA1c) was under control (i.e. HbA1c < 7.0%).

Furthermore, a sensitivity analysis was conducted regarding a threshold haemoglobin level at which the SGLT2i-induced erythrocytosis would be associated with different cardiovascular profiles. To this end, a threshold of haemoglobin > 17.5 g/dL for men and > 17.0 g/dL for women were chosen, respectively, which were 1.0 g/dL above the sex-specific definition of erythrocytosis. The risk period of erythrocytosis and SGLT2i use was further split into 1) SGLT2i use with erythrocytosis, and haemoglobin level exceeding the above cut-off; and 2) SGLT2i use with erythrocytosis, and haemoglobin level > 16.5–≤ 17.5 g/dL (for men) or > 16.0–≤ 17.0 g/dL (for women) for SCCS analysis.

All statistical analyses were performed with the STATA version SE 17.0 (StataCorp LLC). A two-sided significance level of 5% was used in all statistical analyses.

## Results

**Fig. 3:**
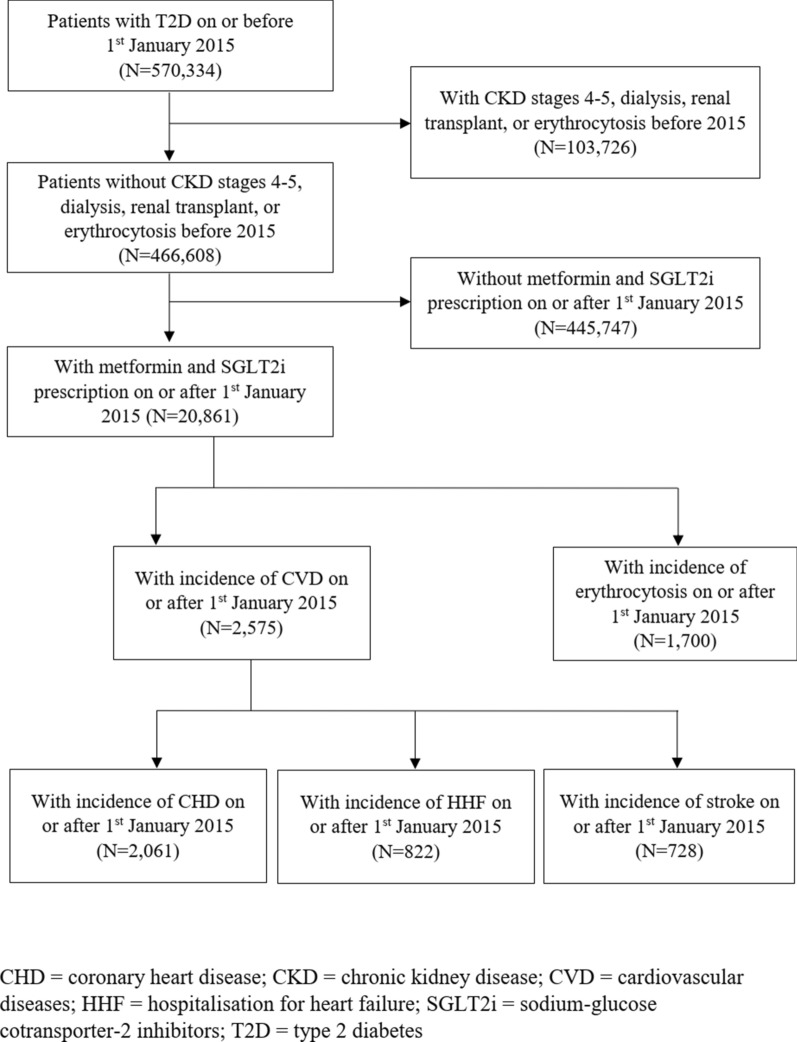
Flowchart of inclusion of eligible patients in this self-controlled case series study. Patients who had received SGLT2i and/or metformin after the diagnosis of type 2 diabetes were included in the current analysis. Those with stage 4–5 chronic kidney disease, dialysis, renal transplant, or erythrocytosis before the start of observation (1st January 2015) were excluded. In total, 20,861 patients with type 2 diabetes and metformin and/or SGLT2i prescription records on or after 1st January 2015 were identified

In total, 20,861 T2D patients with metformin and/or SGLT2i prescription records on or after 1st January 2015 were identified (20,499, 19,629 and 2738 patients included in the metformin-only period, metformin-SGLT2i combination period, and SGLT2i-only period, respectively). A total of 2,575, 2,061, 822, 728, and 1,700 events of composite CVD, CHD, HHF, stroke, and erythrocytosis were observed, respectively (Fig. 1). The inclusion of eligible patients in this SCCS study is shown in Fig. 3, and patient characteristics by event outcomes are listed in Table 1.

**Table 1:** Patient characteristics by event outcomes at the start of observation period

% (n)/mean ± standard deviation (n)	Cardiovascular diseases (N = 2575)	Coronary heart disease (N = 2061)	Hospitalisation for heart failure (N = 822)	Stroke (N = 728)	Erythrocytosis (N = 1700)
*Socio-demographics*					
Sex					
Female	39.3% (1,011)	34.7% (716)	44.2% (363)	41.4% (301)	20.0% (340)
Male	60.7% (1564)	65.3% (1,345)	55.8% (459)	58.7% (427)	80.0% (1360)
Age, year	62.3 ± 9.6 (2575)	62.0 ± 9.5 (2061)	66.2 ± 10.4 (822)	64.2 ± 9.8 (728)	55.4 ± 10.6 (1700)
Smoking status					
Non-smoker	63.2% (1560)	61.0% (1205)	60.6% (471)	62.6% (432)	52.3% (839)
Ex-smoker	19.5% (480)	20.1% (397)	22.7% (176)	22.0% (152)	25.9% (415)
Current smoker	17.3% (428)	18.9% (374)	16.7% (130)	15.4% (106)	21.8% (349)
Alcohol status					
Non-drinker	67.6% (1520)	65.9% (1183)	71.3% (491)	67.6% (420)	56.0% (810)
Ex-drinker	7.7% (174)	8.2% (147)	9.3% (64)	9.7% (60)	9.7% (140)
Social drinker	20.8% (468)	21.9% (394)	16.0% (110)	19.0% (118)	28.8% (417)
Current drinker	3.8% (86)	4.0% (72)	3.5% (24)	3.7% (23)	5.5% (79)
*Clinical characteristics*					
HbA1c, %	7.9 ± 1.5 (2534)	7.9 ± 1.5 (2033)	8.0 ± 1.7 (811)	8.2 ± 1.6 (716)	8.2 ± 1.5 (1674)
Fasting glucose, mmol/L	8.5 ± 2.8 (2442)	8.5 ± 2.9 (1955)	8.6 ± 3.1 (790)	8.9 ± 2.9 (704)	8.9 ± 2.9 (1651)
Serum creatinine, umol/L	77.7 ± 20.8 (2553)	79.2 ± 21.2 (2043)	81.3 ± 25.7 (817)	77.7 ± 20.1 (724)	80.2 ± 21.3 (1691)
Estimated glomerular filtration rate, mL/min/1.73m^2^	87.4 ± 20.5 (1841)	86.2 ± 19.5 (1453)	82.7 ± 22.8 (555)	87.5 ± 22.1 (449)	91.3 ± 21.7 (1030)
Urine albumin to creatinine ratio, mg/mmol	3.7 ± 5.9 (1982)	3.5 ± 5.4 (1566)	5.4 ± 7.5 (599)	3.9 ± 5.6 (519)	3.8 ± 5.9 (1210)
Systolic blood pressure, mmHg	134.4 ± 15.9 (2184)	134.1 ± 16.0 (1737)	136.3 ± 18.4 (657)	135.9 ± 17.2 (574)	133.2 ± 17.2 (1166)
Diastolic blood pressure, mmHg	75.8 ± 10.7 (2184)	76.2 ± 10.7 (1737)	74.1 ± 12.1 (657)	75.9 ± 11.6 (574)	79.4 ± 10.6 (1166)
Low-density lipoprotein cholesterol, mmol/L	2.5 ± 0.8 (2508)	2.5 ± 0.8 (2010)	2.4 ± 0.8 (801)	2.5 ± 0.8 (714)	2.4 ± 0.8 (1667)
Total cholesterol, mmol/L	4.4 ± 1.0 (2519)	4.4 ± 1.0 (2019)	4.3 ± 1.1 (806)	4.4 ± 1.0 (716)	4.3 ± 0.9 (1670)
High-density lipoprotein cholesterol, mmol/L	1.2 ± 0.3 (2513)	1.2 ± 0.3 (2015)	1.2 ± 0.3 (803)	1.2 ± 0.3 (714)	1.1 ± 0.3 (1668)
Total cholesterol to high-density lipoprotein cholesterol ratio	4.0 ± 1.2 (2513)	4.0 ± 1.3 (2,015)	3.9 ± 1.3 (803)	3.9 ± 1.2 (714)	4.0 ± 1.2 (1668)
Triglyceride, mmol/L	1.7 ± 1.4 (2518)	1.7 ± 1.6 (2018)	1.7 ± 1.3 (805)	1.7 ± 1.1 (715)	1.8 ± 1.8 (1670)
Body mass index, kg/m^2^	28.4 ± 54.5 (1878)	28.1 ± 59.0 (1493)	27.8 ± 5.4 (538)	27.9 ± 28.5 (467)	28.6 ± 5.1 (948)
Haemoglobin, g/dL	13.6 ± 1.4 (2220)	13.6 ± 1.4 (1777)	13.3 ± 1.5 (752)	13.4 ± 1.4 (666)	14.6 ± 1.2 (1562)
Haematocrit, L/L	0.40 ± 0.04 (2190)	0.40 ± 0.04 (1757)	0.39 ± 0.04 (747)	0.40 ± 0.04 (659)	0.43 ± 0.03 (1557)
Red blood cell, × 10^12^ /L	4.6 ± 0.6 (2191)	4.7 ± 0.6 (1758)	4.5 ± 0.6 (747)	4.6 ± 0.6 (659)	5.0 ± 0.5 (1557)
Duration of diabetes, year	11.9 ± 7.7 (2575)	12.1 ± 8.0 (2061)	12.6 ± 8.1 (822)	12.3 ± 7.5 (728)	10.3 ± 6.9 (1700)
Charlson Comorbidity Index	6.0 ± 1.3 (2575)	6.1 ± 1.3 (2061)	7.0 ± 1.6 (822)	6.5 ± 1.5 (728)	5.7 ± 1.5 (1700)
Severe hypoglycaemia (within 1 year before start of observation)	2.8% (71)	2.8% (57)	4.4% (36)	5.2% (38)	3.7% (62)
*Use of medications (ever before start of observation)*					
Insulin	28.0% (721)	28.4% (585)	33.7% (277)	34.3% (250)	38.9% (661)
Sulfonylurea	83.7% (2156)	82.6% (1702)	84.6% (695)	87.5% (637)	83.7% (1422)
Thiazolidinedione	4.5% (115)	4.4% (91)	4.9% (40)	4.5% (33)	7.5% (127)
Dipeptidyl peptidase-4 inhibitors	17.4% (447)	16.5% (339)	20.0% (164)	22.8% (166)	25.8% (438)
Glucagon-like peptide-1 receptor agonists	0.3% (7)	0.3% (6)	0.2% (2)	0.4% (3)	1.5% (26)
Alpha-glucosidase inhibitors	6.2% (159)	6.3% (130)	9.0% (74)	8.1% (59)	6.9% (117)
Meglitinide	0.0% (0)	0.0% (0)	0.0% (0)	0.0% (0)	0.0% (0)
Antihypertensive drugs					
Angiotensin-converting enzyme inhibitors / angiotensin receptor blockers	71.8% (1849)	71.5% (1474)	82.2% (676)	77.9% (567)	71.2% (1210)
Beta blocker	44.4% (1142)	44.7% (922)	62.3% (512)	55.0% (400)	47.1% (800)
Calcium channel blockers	64.0% (1649)	63.9% (1316)	74.7% (614)	65.3% (475)	55.6% (945)
Diuretics	29.8% (767)	28.7% (592)	46.5% (382)	36.4% (265)	27.3% (464)
Others	18.0% (464)	18.3% (378)	27.5% (226)	20.1% (146)	12.6% (214)
Lipid-lowering agents	68.9% (1774)	72.3% (1490)	75.9% (624)	69.2% (504)	73.9% (1257)
Antiplatelet / anticoagulants	25.1% (647)	31.1% (640)	50.1% (412)	40.9% (298)	37.4% (635)
Testosterone (for male only)	0.3% (5)	0.5% (6)	0.0% (0)	0.5% (2)	0.6% (8)
Hormone therapy (for female only)	8.2% (83)	9.1% (65)	5.8% (21)	8.6% (26)	16.8% (57)
*Type of SGLT2i used*					
Canagliflozin	0.1% (2)	0.1% (2)	0.0% (0)	0.1% (1)	0.4% (6)
Dapagliflozin	28.7% (738)	26.7% (551)	28.1% (231)	34.3% (250)	37.6% (639)
Empagliflozin	71.3% (1836)	73.3% (1510)	72.0% (592)	65.5% (477)	62.0% (1054)
Ertugliflozin	0.0% (0)	0.0% (0)	0.0% (0)	0.0% (0)	0.1% (1)

HbA1c: glycated haemoglobin; SGLT2i: sodium-glucose cotransporter-2 inhibitors

**Fig. 4:**
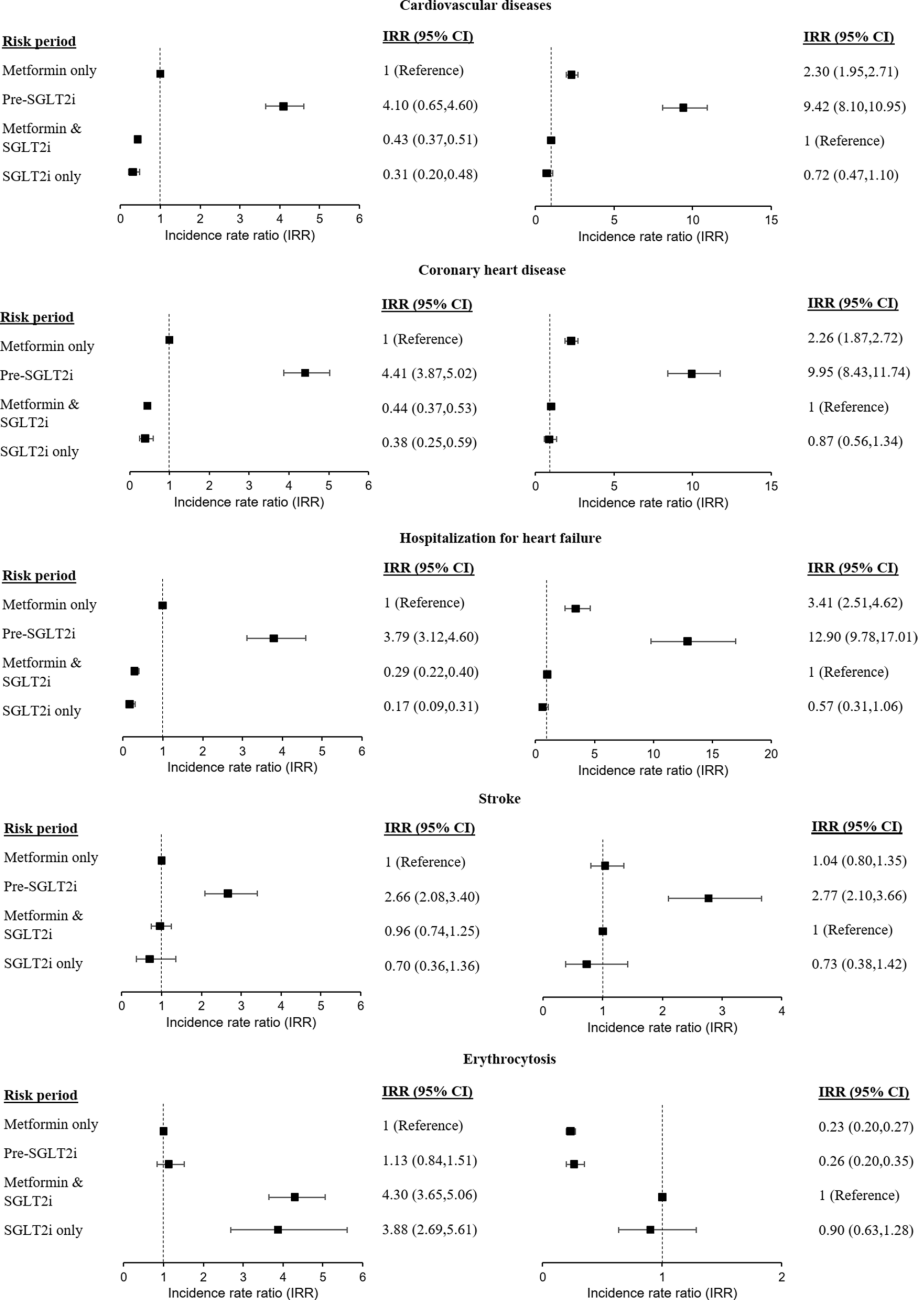
Forest plot of incidence rate ratios of study outcomes by exposure of metformin and SGLT2i. The incidence rate ratio of study outcomes between periods of (i) metformin use without SGLT2i; (ii) pre-SGLT2i (three months before each initiation of SGLT2i); (iii) SGLT2i use alone period; and (iv) combined use of metformin and SGLT2i period, were estimated using conditional Poisson regression model with an offset for the length of the risk period, with adjustment of time-varying factors (age and the use of other anti-diabetic agents [sulfonylureas, thiazolidinediones, dipeptidyl peptidase-4 inhibitors, GLP1 receptor agonists, and insulin])

Compared to the ‘baseline period’ of metformin only, the IRR and 95%CI of patients who had composite CVD, CHD, HHF, stroke, and erythrocytosis in different risk periods are presented in Fig. 4 and Additional file 1: Table S2. Subgroup analyses by individual SGLT2i, i.e. dapagliflozin and empagliflozin, are shown in Additional file 1: Tables S3 and S4, respectively.

### Incident cardiovascular diseases

Since T2D patients with established CVD are the optimal candidates for SGLT2 initiation, it was expected that the risk of composite CVD was increased in the pre-SGLT2i period. SGLT2i use was associated with significant reduction in CVD risk. This reduction in CVD risk was similar with or without concomitant metformin, as demonstrated by the IRR of 0.43 and 0.31 in the periods of combination therapy and SGLT2i only compared to baseline (both p < 0.001). Similar results were noted on sensitivity analyses for dapagliflozin and empagliflozin use. Furthermore, the comparison of IRR between the periods of combination therapy and SGLT2i only did not reveal a significant difference (p = 0.127), suggesting no significant influence of concomitant metformin use on SGLT2i-associated cardiovascular benefits. Results for the risks of CHD and HHF were consistently seen as in composite CVD.

Similar to that observed in the above CVD outcomes, the incidence of stroke was significantly higher in the pre-SGLT2i period. In contrast, SGLT2i use was neutral on the risk of stroke compared to ‘baseline period’ of metformin only, which was consistent in sensitivity analyses for dapagliflozin and empagliflozin use. There was also no difference on the risk of stroke regardless of the presence of concomitant metformin prescription.

### Erythrocytosis

The incidence rate of erythrocytosis per 100 person-years were 0.75 for the ‘baseline period’ of metformin only, and 3.06–3.27 during SGLT2i use. SGLT2i use was associated with higher incidences of erythrocytosis (IRR = 3.88–4.30, p < 0.001), regardless of concomitant metformin prescription, and consistently observed in sensitivity analyses for dapagliflozin and empagliflozin use.

### Impact of addition of SGLT2i on the CVD risks of metformin users who had good glycaemic control (HbA1c < 7.0%)

We demonstrated that addition of SGLT2i in this scenario was also associated with lower CVD risks, consistent with the main analysis (Additional file 1: Table S5). Nonetheless, HHF did not reach statistical significance in this subgroup analysis as the event rates in the metformin-SGLT2i combination and SGLT2i monotherapy exposure periods were rather small.

### Association between erythrocytosis and CVD risks

**Fig. 5:**
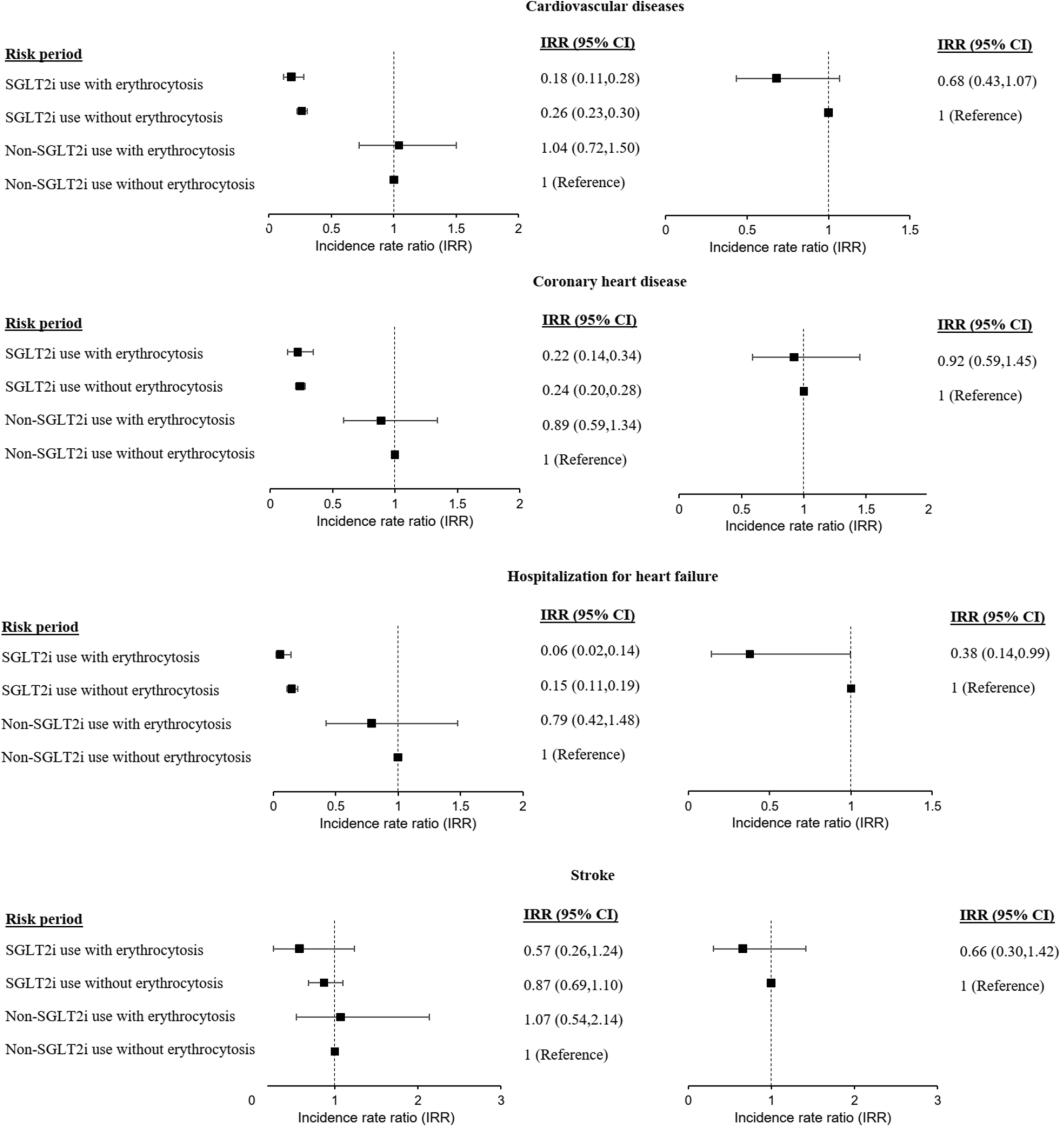
Forest plot of incidence rate ratios of cardiovascular outcomes by SGLT2i exposure and erythrocytosis. The incidence rate ratio of cardiovascular outcomes between periods of (i) SGLT2i exposure with erythrocytosis; (ii) SGLT2i exposure without erythrocytosis; (iii) absence of SGLT2i exposure with erythrocytosis; and (iv) absence of SGLT2i exposure without erythrocytosis, were estimated using conditional Poisson regression model with an offset for the length of the risk period, with adjustment of time-varying factors (age and the use of other anti-diabetic agents [sulfonylureas, thiazolidinediones, dipeptidyl peptidase-4 inhibitors, GLP1 receptor agonists, and insulin])

Compared to the reference period of non-SGLT2i use without erythrocytosis, erythrocytosis in non-SGLT2i users did not have higher risk of CVD (Fig. 5 and Additional file 1: Table S6). On the other hand, SGLT2i use was associated with risk reduction of composite CVD, CHD and HHF. Among SGLT2i users who developed erythrocytosis, a reduction in the risk of HHF (IRR = 0.38, p = 0.049) was observed comparing to SGLT2i users who did not develop erythrocytosis. In the sensitivity analysis of the haemoglobin threshold for SGLT2i-associated cardiovascular benefits, we noted that reduction in the risk of HHF was still present (IRR = 0.33, p = 0.036) when haemoglobin was within 1.0 g/dL above the sex-specific cut-off, but not when the haemoglobin level was elevated beyond that (Additional file 1: Table S7).

## Discussion

Using real-world data in a population-based SCCS study of T2D patients, we demonstrated that SGLT2i use was associated with reduction in CVD risks – composite CVD, CHD, and HHF – compared with baseline metformin use. Moreover, the SGLT2i-associated cardiovascular benefits were not influenced by concomitant metformin use. Our real-world data clarified the concern about the attenuation of SGLT2i-associated cardiovascular benefits with metformin use, boosting the confidence of diabetes care providers in initiating SGLT2i for suitable candidates. Furthermore, the incidence of erythrocytosis was significantly higher with SGLT2i use. Interestingly, erythrocytosis during SGLT2i exposure did not attenuate its cardiovascular benefits. Given the risk reduction in HHF among SGLT2i users with erythrocytosis, erythrocytosis may be a surrogate marker of this SGLT2i-associated cardiovascular benefit. Our results should raise clinicians’ awareness of SGLT2i-associated erythrocytosis: while SGLT2i are indicated for glycaemic control and cardiovascular benefits in T2D patients, clinicians should monitor SGLT2i users who develop erythrocytosis and exclude sinister causes such as polycythaemia vera (PV).

Our results have clarified concerns about the attenuation of SGLT2i-associated cardiovascular benefits by metformin use. As metformin activates AMPK but not primarily SIRT1, gluconeogenesis and ketogenesis are inhibited among metformin users, in addition to reducing haematocrit and increasing uric acid levels; these are directly opposite to the effects of SGLT2i, and possibly attenuating SGLT2i-associated benefits on HF [29]. Our results did not demonstrate the attenuation of SGLT2i-associated cardiovascular benefits by metformin. Although a pooled meta-analysis of cardiovascular outcomes trials of SGLT2i has identified potential attenuation of cardiovascular benefits with concomitant metformin use, the apparent attenuation of SGLT2i benefits on MACE by metformin might have been confounded by indication bias or imbalances in the patient characteristics between metformin users and non-users [30]. By adopting the SCCS design, we were able to control for time-invariant confounders and the use of other antidiabetic agents. Nonetheless, as most patients in our cohort were prescribed either empagliflozin or dapagliflozin, we could not examine other SGLT2i such as canagliflozin. The partial mechanistic overlap of AMPK activation between SGLT2i and metformin attenuating SGLT2i-associated cardiovascular benefits might depend on the degree of AMPK activation by individual SGLT2i. For instance, a significant interaction between canagliflozin and metformin on the risk of HHF or cardiovascular death has been attributed to the more direct and larger effect of this SGLT2i on AMPK activation [22, 23, 31, 32]. Further research is warranted to explore the clinical significance of any interaction between other SGLT2i and metformin in specific CVD outcomes [22].

In addition to providing reassurance that metformin does not attenuate the SGLT2i-associated cardiovascular benefits, our results also shed light onto whether metformin should be continued when SGLT2i is introduced. The comparison between metformin-SGLT2i combination period and SGLT2i only period showed comparable risks of all cardiovascular outcomes (all p > 0.05). As metformin has high glycaemic efficacy, with potential for modest weight loss and low treatment cost, our current evidence suggests that metformin should be continued when SGLT2i is introduced.

In line with case reports of SGLT2i-induced erythrocytosis [17, 33, 34] and recent studies identifying increases in haematocrit, haemoglobin or erythrocyte concentration as the most important mediator of SGLT2i in reducing the risks of HF and cardiovascular death among T2D patients [15, 16, 31, 32], our findings demonstrated that SGLT2i use (alone or in combination with metformin) was associated with significantly lower risks of composite CVD, CHD and HHF in T2D patients; and concomitantly, an increased rate of erythrocytosis. Also, SGLT2i users who developed erythrocytosis had a lower risk of HHF compared to those who did not, which was not seen in non-SGLT2i users who developed erythrocytosis. Several mechanisms have been proposed to illustrate this statistical mediation. First, the induction of a fasting-like transcriptional paradigm by SGLT2 inhibition may activate several nutrient deprivation pathways, namely the upregulation of SIRT1 and HIF-2α that stimulate ketogenesis and erythrocytosis, respectively, in addition to that of AMPK [29, 32]. Downstream effects of activating these regulators include the promotion of autophagy and cellular health, improvement of mitochondrial function, and reduction of oxidative stress and inflammation, which may facilitate cardio-protection and mitigate cardiac injury [29, 32]. Such molecular reprogramming appears to be a system-wide effect induced by glycosuria and perceived nutrient deprivation associated with SGLT2 inhibition, despite an absence of SGLT2 expression in the heart and other organs [29, 32]. Second, elevated haematocrit observed in T2D patients may be mediated via an alleviation of renal stress and glucotoxicity, hence restoration of erythropoietin production; alongside the correction of sympathetic hyperactivity and subsequent prevention of HF [35, 36]. Apart from being proposed as a surrogate marker of reducing metabolic stress [35], the elevated haematocrit could have also improved oxygen delivery to the myocardium, and possibly cardiac function [14, 37, 38]. Third, SGLT2i use has been associated with enhanced erythropoiesis via increased mobilisation and utilisation of iron, as reflected by reduced concentrations of hepcidin and ferritin [39]. This is a crucial point in patients with heart failure with reduced ejection fraction and iron deficiency in whom we should consider adding iron (III) carboxymaltose to therapy regardless of the presence of anaemia [40–42]. Whether iron (III) carboxymaltose could increase the SGLT2i-associated benefits by providing substance for erythropoiesis remains to be elucidated. Notably, SGLT2i seem to promote erythropoietin secretion and increase haematocrit on top of RAAS blockade (which is haematocrit-lowering), potentially contributing to cardiorenal benefits in those already taking RAAS inhibitors [36] (i.e. most of our patients).

Our study provided useful information on the incidence of SGLT2i-associated erythrocytosis in a population-based cohort—around 3 per 100 person-years. We have also characterised the cardiovascular outcomes of patients who developed SGLT2i-associated erythrocytosis. In contrast to non-SGLT2i-associated polycythaemia that has been associated with increased risks of MACE [18], SGLT2i-associated erythrocytosis does not appear to attenuate the cardiovascular benefits of SGLT2i, hence phlebotomy might not be necessary in this case [17, 32]. This may be explained by the fact that SGLT2i activates both SIRT1 and AMPK which are associated with a fasting transcriptional paradigm. This brings about antioxidant and anti-inflammatory effects, and consequently cardio-protection [43]. Erythrocytosis is only one of the downstream effects from SIRT1 activation. In fact, we even observed a lower risk of HHF in SGLT2i users who developed erythrocytosis compared to SGLT2i users who did not. Our sensitivity analysis of the threshold haemoglobin level for SGLT2i-associated cardiovascular benefits demonstrated that mild erythrocytosis (haemoglobin level elevated within 1.0 g/dL above the sex-specific cut-off) during SGLT2i use was even associated with further cardiovascular benefits in HHF. While our current study did not reveal an increase in cardiovascular risks with even higher haemoglobin, this could likely be due to the relatively small number of events in that subgroup. Taken together, these results could mean that while clinicians should monitor SGLT2i users who develop erythrocytosis and exclude sinister causes such as PV, clinicians could probably tolerate mild SGLT2i-asociated erythrocytosis for its associated cardiovascular benefits. Further systematic assessment of SGLT2i-associated erythrocytosis will shed light on the haemoglobin threshold and the validity of using erythrocytosis as a potential marker of SGLT2i users who may derive cardiovascular benefits.

Our study revealed that SGLT2i use (with or without concomitant metformin) was not associated with an increased risk of stroke despite a higher incidence of erythrocytosis. While significant benefits of SGLT2i on HHF and cardiovascular death have been robustly shown in randomised trials, they seem to exert a neutral effect or even a trend towards higher risk of stroke than placebo [44, 45]. Further analysis of the EMPA-REG OUTCOME trial has identified a numeric difference in stroke events occurring ˃90 days following the last intake of empagliflozin; yet the risk of cerebrovascular events was not significantly increased, and a causal association with this SGLT2i was deemed unlikely [46]. Recently, a retrospective cohort study conducted in Taiwan has compared the effects of first-line SGLT2i and metformin in T2D patients, and concluded that the former was associated with significantly lower risks of acute coronary syndrome, HHF, and all-cause mortality; however, the risk of ischaemic stroke was higher in SGLT2i than metformin users [47]. Concerns have been raised regarding any association between the elevated haematocrit seen with SGLT2i and a lack of benefits (or even potential harm) of this drug class on stroke, as increases in haematocrit and blood viscosity have been associated with higher incidences of cerebrovascular diseases, especially ischaemic stroke [44, 48]. On the contrary, it has been argued that such association between elevated haematocrit and stroke could be confounded by other cardiovascular risk factors, namely smoking and hypertension [49, 50]; as well as the nature of erythrocytosis, where substantially higher incidences of thrombotic or cerebrovascular events have been observed in patients with PV compared to those with secondary erythrocytosis [49–51]. Besides, an increased risk of stroke was not evident among SGLT2i users with the largest increments in haematocrit in the EMPA-REG OUTCOME trial [46]; and in another study, no significant differences in haematocrit level were noted between patients with or without cerebrovascular diseases [51]. Using the SCCS design, our study has taken care of potential confounders such as smoking and hypertension, and provided reassuring results that were generally in line with the current literature. Possible antioxidant and anti-inflammatory effects downstream of AMPK and SIRT1 activation may explain the neutral impact of SGLT2i use on stroke.

Our study provided additional clinical evidence supporting the introduction of SGLT2i to metformin users even when glycaemic management is under control, for its cardiovascular benefits [8–11]. Adopting the SCCS study design, fixed and time-invariant confounders have been accounted for, in addition to addressing immortal time bias. Furthermore, our study has included a pre-SGLT2i exposure period to acknowledge the probability of drug exposure by indications, and adopted an extended version of SCCS to adjust for censoring by outcome. Nevertheless, there were several limitations in this study. First, the number of cases was relatively small with short follow-up periods, especially for the exposure period of SGLT2i use without metformin. Second, information on *JAK2* mutation and serum erythropoietin level was not available for the exclusion of PV in our patients. Third, subgroup analyses by individual SGLT2i could only be conducted on dapagliflozin and empagliflozin, so our main results might not be generalisable to canagliflozin and other SGLT2i given their very limited use in this study. Fourth, echocardiographic parameters were not available from the database. Lastly, as only patients with both drug exposure and outcomes of interest were included in this study by the nature of the SCCS method, selection bias could not be entirely excluded. Another limitation of the SCCS approach was that changes in other clinical characteristics of patients that would have an impact on the clinical decision making, and hence related to the study exposure and/or outcomes, might not be captured or fully accounted for even with the inclusion of pre-SGLT2i period.

## Conclusion

Our real-world data suggested that SGLT2i-associated cardiovascular benefits were not attenuated by metformin use. Although SGLT2i use was associated with increased incidence of erythrocytosis, the occurrence of erythrocytosis did not attenuate SGLT2i-associated cardiovascular benefits. There were potentially further cardiovascular benefits among SGLT2i users who developed erythrocytosis. Further studies into the role of erythrocytosis as a potential surrogate marker of SGLT2i-associated reduction in the risk of HHF are warranted.

## Permissions information

The authors do hereby declare that all illustrations and figures in the manuscript are entirely original and do not require reprint permission.

## Supplementary Information

**Additional file 1: Table S1.** Definition of disease and outcome event diagnoses. **Table S2.** Incidence rate ratios of event outcomes in different exposure periods of metformin and SGLT2i use. **Table S3.** Incidence rate ratios of cardiovascular diseases, coronary heart disease, hospitalisation for heart failure, stroke, and erythrocytosis in different exposure periods of metformin and dapagliflozin use. **Table S4.** Incidence rate ratios of cardiovascular diseases, coronary heart disease, hospitalisation for heart failure, stroke, and erythrocytosis in different exposure periods of metformin and empagliflozin use. **Table S5.** Incidence rate ratios of cardiovascular diseases, coronary heart disease, hospitalisation for heart failure, stroke, and erythrocytosis in different exposure periods of metformin and SGLT2i use for patients who achieved targeted glycaemic control (HbA1c < 7%) when initiated SGLT2i. **Table S6.** Incidence rate ratios of event outcomes in different risk periods by SGLT2i exposure and erythrocytosis. **Table S7.** Incidence rate ratios of cardiovascular diseases, coronary heart disease, hospitalisation for heart failure, and stroke in different risk periods by any exposure to SGLT2i and the development of erythrocytosis, with sex-specific cut-off for haemoglobin.

## Data Availability

The data that support the findings of this study were provided by the Hong Kong Hospital Authority. Restrictions apply to the availability of these data, which were used under license for the current study, and so are not publicly available.

## Abbreviations

ADA: American Diabetes Association
AMPK: AMP-activated protein kinase
CHD: Coronary heart disease
CI: Confidence interval
CVD: Cardiovascular diseases
HF: Heart failure
HHF: Hospitalisation for heart failure
HIF: Hypoxia-inducible factors
ICD-9-CM: International Statistical Classification of Diseases and Related Health Problems, 9th Revision, Clinical Modification
ICD-10-CM: International Statistical Classification of Diseases and Related Health Problems, 10th Revision, Clinical Modification
ICPC-2: International Classification of Primary Care, Version 2
IR: Incidence rate
IRR: Incidence rate ratio
MACE: Major adverse cardiovascular events
PV: Polycythemia vera
RAAS: Renin–angiotensin–aldosterone system
SCCS: Self-controlled case series
SGLT2i: Sodium-glucose cotransporter-2 inhibitors
SIRT-1: Sirtuin-1
T2D: Type 2 diabetes

## References

[1] Einarson TR, Acs A, Ludwig C, Panton UH (2018). Prevalence of cardiovascular disease in type 2 diabetes: a systematic literature review of scientific evidence from across the world in 2007–2017. Cardiovasc Diabetol.

[2] Das SR, Everett BM, Birtcher KK, Brown JM, Januzzi JL, Kalyani RR (2020). 2020 Expert consensus decision pathway on novel therapies for cardiovascular risk reduction in patients with type 2 diabetes: a report of the American College of Cardiology Solution Set Oversight Committee. J Am Coll Cardiol.

[3] Mahaffey KW, Jardine MJ, Bompoint S, Cannon CP, Neal B, Heerspink HJL (2019). Canagliflozin and cardiovascular and renal outcomes in type 2 diabetes mellitus and chronic kidney disease in primary and secondary cardiovascular prevention groups. Circulation.

[4] Mahaffey KW, Neal B, Perkovic V, de Zeeuw D, Fulcher G, Erondu N (2018). Canagliflozin for Primary and Secondary Prevention of Cardiovascular Events. Circulation.

[5] Neuen BL, Ohkuma T, Neal B, Matthews DR, de Zeeuw D, Mahaffey KW (2018). Cardiovascular and renal outcomes with canagliflozin according to baseline kidney function. Circulation.

[6] Wanner C, Inzucchi SE, Zinman B, Koitka-Weber A, Mattheus M, George JT (2020). Consistent effects of empagliflozin on cardiovascular and kidney outcomes irrespective of diabetic kidney disease categories: Insights from the EMPA-REG OUTCOME trial. Diabetes Obes Metab.

[7] Zannad F, Ferreira JP, Pocock SJ, Anker SD, Butler J, Filippatos G (2020). SGLT2 inhibitors in patients with heart failure with reduced ejection fraction: a meta-analysis of the EMPEROR-Reduced and DAPA-HF trials. The Lancet.

[8] American Diabetes Association Professional Practice Committee (2022). 9. Pharmacologic approaches to glycemic treatment: standards of medical care in diabetes—2022. Diabetes Care.

[9] Cosentino F, Grant PJ, Aboyans V, Bailey CJ, Ceriello A, Delgado V (2020). 2019 ESC Guidelines on diabetes, pre-diabetes, and cardiovascular diseases developed in collaboration with the EASD: The Task Force for diabetes, pre-diabetes, and cardiovascular diseases of the European Society of Cardiology (ESC) and the European Association for the Study of Diabetes (EASD). Eur Heart J.

[10] Garber AJ, Handelsman Y, Grunberger G, Einhorn D, Abrahamson MJ, Barzilay JI (2020). Consensus statement by the American Association of clinical endocrinologists and American College of Endocrinology on the comprehensive type 2 diabetes management algorithm—2020 executive summary. Endocr Pract.

[11] Visseren FLJ, Mach F, Smulders YM, Carballo D, Koskinas KC, Bäck M (2021). 2021 ESC guidelines on cardiovascular disease prevention in clinical practice: Developed by the Task Force for cardiovascular disease prevention in clinical practice with representatives of the European Society of Cardiology and 12 medical societies with the special contribution of the European Association of Preventive Cardiology (EAPC). Eur Heart J.

[12] Margonato D, Galati G, Mazzetti S, Cannistraci R, Perseghin G, Margonato A (2021). Renal protection: a leading mechanism for cardiovascular benefit in patients treated with SGLT2 inhibitors. Heart Fail Rev.

[13] Tuttle KR, Brosius FC, Cavender MA, Fioretto P, Fowler KJ, Heerspink HJL (2020). SGLT2 inhibition for CKD and cardiovascular disease in type 2 diabetes: report of a scientific workshop sponsored by the national kidney foundation. Diabetes.

[14] Zelniker TA, Braunwald E (2020). Mechanisms of cardiorenal effects of sodium-glucose cotransporter 2 inhibitors: JACC state-of-the-art review. J Am Coll Cardiol.

[15] Inzucchi SE, Zinman B, Fitchett D, Wanner C, Ferrannini E, Schumacher M (2017). How does empagliflozin reduce cardiovascular mortality? insights from a mediation analysis of the EMPA-REG OUTCOME Trial. Diabetes Care.

[16] Li J, Woodward M, Perkovic V, Figtree GA, Heerspink HJL, Mahaffey KW (2020). Mediators of the effects of canagliflozin on heart failure in patients with type 2 diabetes. JACC Heart Fail..

[17] Gangat N, Szuber N, Alkhateeb H, Al-Kali A, Pardanani A, Tefferi A (2021). JAK2 wild-type erythrocytosis associated with sodium-glucose cotransporter 2 inhibitor therapy. Blood.

[18] Kim I-S, Lee BK, Yang P-S, Joung B, Kim J-Y (2020). Sex-based approach for the clinical impact of polycythaemia on cardiovascular outcomes in the general population. Eur J Prev Cardiol.

[19] Rådholm K, Figtree G, Perkovic V, Solomon SD, Mahaffey KW, de Zeeuw D (2018). Canagliflozin and heart failure in type 2 diabetes mellitus. Circulation.

[20] Inzucchi SE, Fitchett D, Jurišić-Eržen D, Woo V, Hantel S, Janista C (2020). Are the cardiovascular and kidney benefits of empagliflozin influenced by baseline glucose-lowering therapy?. Diabetes Obes Metab.

[21] Cahn A, Wiviott SD, Mosenzon O, Murphy SA, Goodrich EL, Yanuv I (2021). Cardiorenal outcomes with dapagliflozin by baseline glucose-lowering agents: post hoc analyses from DECLARE-TIMI 58. Diabetes Obes Metab.

[22] Packer M (2020). Are the benefits of SGLT2 inhibitors in heart failure and a reduced ejection fraction influenced by background therapy? Expectations and realities of a new standard of care. Eur Heart J.

[23] Packer M (2020). Does metformin interfere with the cardiovascular benefits of SGLT2 inhibitors? Questions about its role as the cornerstone of diabetes treatment. Am J Med.

[24] Pillai AA, Fazal S, Babiker HM (2021). Polycythemia.

[25] Whitaker HJ, Farrington CP, Spiessens B, Musonda P (2006). Tutorial in biostatistics: the self-controlled case series method. Stat Med.

[26] Petersen I, Douglas I, Whitaker H (2016). Self controlled case series methods: an alternative to standard epidemiological study designs. BMJ.

[27] Suissa S (2018). Reduced mortality with sodium-glucose cotransporter-2 inhibitors in observational studies: avoiding immortal time bias. Circulation.

[28] Suissa S (2018). Lower risk of death with SGLT2 inhibitors in observational studies: real or bias?. Diabetes Care.

[29] Packer M (2020). Cardioprotective effects of sirtuin-1 and its downstream effectors. Circ Heart Fail.

[30] Singh AK, Singh R (2021). Does background metformin therapy influence the cardiovascular outcomes with SGLT-2 inhibitors in type 2 diabetes?. Diabetes Res Clin Pract.

[31] Packer M (2020). Autophagy stimulation and intracellular sodium reduction as mediators of the cardioprotective effect of sodium–glucose cotransporter 2 inhibitors. Eur J Heart Fail.

[32] Packer M (2021). Critical examination of mechanisms underlying the reduction in heart failure events with SGLT2 inhibitors: identification of a molecular link between their actions to stimulate erythrocytosis and to alleviate cellular stress. Cardiovasc Res.

[33] Chin-Yee B, Solh Z, Hsia C (2020). Erythrocytosis induced by sodium-glucose cotransporter-2 inhibitors. Can Med Assoc J.

[34] Gupta R, Gupta A, Shrikhande M, Tyagi K, Ghosh A, Misra A (2020). Marked erythrocytosis during treatment with sodium glucose cotransporter-2 inhibitors-report of two cases. Diabetes Res Clin Pract.

[35] Sano M, Goto S (2019). Possible mechanism of hematocrit elevation by sodium glucose cotransporter 2 inhibitors and associated beneficial renal and cardiovascular effects. Circulation.

[36] Marathias KP, Lambadiari VA, Markakis KP, Vlahakos VD, Bacharaki D, Raptis AE (2020). Competing effects of renin angiotensin system blockade and sodium-glucose cotransporter-2 inhibitors on erythropoietin secretion in diabetes. Am J Nephrol.

[37] Mazer CD, Hare GMT, Connelly PW, Gilbert RE, Shehata N, Quan A (2020). Effect of empagliflozin on erythropoietin levels, iron stores, and red blood cell morphology in patients with type 2 diabetes mellitus and coronary artery disease. Circulation.

[38] Gaborit B, Ancel P, Abdullah AE, Maurice F, Abdesselam I, Calen A (2021). Effect of empagliflozin on ectopic fat stores and myocardial energetics in type 2 diabetes: the EMPACEF study. Cardiovasc Diabetol.

[39] Ghanim H, Abuaysheh S, Hejna J, Green K, Batra M, Makdissi A (2020). Dapagliflozin suppresses hepcidin and increases erythropoiesis. J Clin Endocrinol Metab.

[40] Heidenreich PA, Bozkurt B, Aguilar D, Allen LA, Byun JJ, Colvin MM (2022). AHA/ACC/HFSA guideline for the management of heart failure: a report of the American College of Cardiology/American Heart Association Joint Committee on clinical practice guidelines. Circulation.

[41] McDonagh TA, Metra M, Adamo M, Gardner RS, Baumbach A, Böhm M (2021). ESC Guidelines for the diagnosis and treatment of acute and chronic heart failure: Developed by the Task Force for the diagnosis and treatment of acute and chronic heart failure of the European Society of Cardiology (ESC) With the special contribution of the Heart Failure Association (HFA) of the ESC. Eur Heart J.

[42] Sawicki KT, Ardehali H (2021). Intravenous iron therapy in heart failure with reduced ejection fraction: tackling the deficiency. Circulation.

[43] Packer M (2020). SGLT2 inhibitors produce cardiorenal benefits by promoting adaptive cellular reprogramming to induce a state of fasting mimicry: a paradigm shift in understanding their mechanism of action. Diabetes Care.

[44] Imprialos KP, Boutari C, Stavropoulos K, Doumas M, Karagiannis AI (2017). Stroke paradox with SGLT-2 inhibitors: a play of chance or a viscosity-mediated reality?. J Neurol Neurosurg Psychiatry.

[45] Zelniker TA, Wiviott SD, Raz I, Im K, Goodrich EL, Bonaca MP (2019). SGLT2 inhibitors for primary and secondary prevention of cardiovascular and renal outcomes in type 2 diabetes: a systematic review and meta-analysis of cardiovascular outcome trials. Lancet.

[46] Zinman B, Inzucchi SE, Lachin JM, Wanner C, Fitchett D, Kohler S (2017). Empagliflozin and cerebrovascular events in patients with type 2 diabetes mellitus at high cardiovascular risk. Stroke.

[47] Chen T-H, Li Y-R, Chen S-W, Lin Y-S, Sun C-C, Chen D-Y (2020). Sodium-glucose cotransporter 2 inhibitor versus metformin as first-line therapy in patients with type 2 diabetes mellitus: a multi-institution database study. Cardiovasc Diabetol.

[48] Yang R, Wang A, Ma L, Su Z, Chen S, Wang Y (2018). Hematocrit and the incidence of stroke: a prospective, population-based cohort study. Ther Clin Risk Manag.

[49] Gordeuk VR, Key NS, Prchal JT (2019). Re-evaluation of hematocrit as a determinant of thrombotic risk in erythrocytosis. Haematologica.

[50] Nguyen E, Harnois M, Busque L, Sirhan S, Assouline S, Chamaki I (2021). Phenotypical differences and thrombosis rates in secondary erythrocytosis versus polycythemia vera. Blood Cancer J.

[51] Bhat V, Gs T, Rao SS, Sarma GRK, Deepalam SK (2022). Clinical and radiological profile of cerebrovascular disease in polycythemia: analysis of neurologic manifestations from a Tertiary Center in South India. J Stroke Cerebrovasc Dis.

