# Supplementary material for: Cardiovascular benefits of SGLT2 inhibitors in type 2 diabetes, interaction with metformin and role of erythrocytosis: a self-controlled case series study
Source: Cardiovasc Diabetol. 2022 Jun 3;21:92. doi: 10.1186/s12933-022-01520-w (PMC9166572; doi:10.1186/s12933-022-01520-w)
Supplement: Supplementary file 1 — Additional file 1: Table S1. Definition of disease and outcome event diagnoses. Table S2. Incidence rate ratios of event outcomes in different exposure periods of metformin and SGLT2i use. Table S3. Incidence rate ratios of cardiovascular diseases, coronary heart disease, hospitalisation for heart failure, stroke, and erythrocytosis in different exposure periods of metformin and dapagliflozin use. Table S4. Incidence rate ratios of cardiovascular diseases, coronary heart disease, hospitalisation for heart failure, stroke, and erythrocytosis in different exposure periods of metformin and empagliflozin use. Table S5. Incidence rate ratios of cardiovascular diseases, coronary heart disease, hospitalisation for heart failure, stroke, and erythrocytosis in different exposure periods of metformin and SGLT2i use for patients who achieved targeted glycaemic control (HbA1c < 7%) when initiated SGLT2i. Table S6. Incidence rate ratios of event outcomes in different risk periods by SGLT2i exposure and erythrocytosis. Table S7. Incidence rate ratios of cardiovascular diseases, coronary heart disease, hospitalisation for heart failure, and stroke in different risk periods by any exposure to SGLT2i and the development of erythrocytosis, with sex-specific cut-off for haemoglobin. [file 12933_2022_1520_MOESM1_ESM.pdf]

Additional File

Content:

Table S1. Definition of disease and outcome event diagnoses

Table S2. Incidence rate ratios of event outcomes in different exposure periods of metformin and SGLT2i use

Table S3. Incidence rate ratios of cardiovascular diseases, coronary heart disease, hospitalisation for heart failure, stroke, and erythrocytosis in different exposure periods of metformin and dapagliflozin use

Table S4. Incidence rate ratios of cardiovascular diseases, coronary heart disease, hospitalisation for heart failure, stroke, and erythrocytosis in different exposure periods of metformin and empagliflozin use

Table S5. Incidence rate ratios of cardiovascular diseases, coronary heart disease, hospitalisation for heart failure, stroke, and erythrocytosis in different exposure periods of metformin and SGLT2i use for patients who achieved targeted glycaemic control (HbA1c<7%) when initiated SGLT2i

Table S6. Incidence rate ratios of event outcomes in different risk periods by SGLT2i exposure and erythrocytosis

Table S7. Incidence rate ratios of cardiovascular diseases, coronary heart disease, hospitalisation for heart failure, and stroke in different risk periods by any exposure to SGLT2i and the development of erythrocytosis, with sex-specific cut-off for haemoglobin

Table S1. Definition of disease and outcome event diagnoses

Event	ICPC-2	ICD-9-CM	ICD-10-CM for mortality	Clinical parameters
Type 2 diabetes mellitus	T90	250.x0; 250.x2	NA	NA
Coronary heart disease	K74-K76	410.x-414.9; V45.81	I20.x-I25.x;	NA
Hospitalisation for heart failure	K77	428.x	I50.x	NA
Stroke	K89-K90	430.x-432.9; 433.01; 433.11; 433.21; 433.31; 433.81; 433.91; 434.01; 434.11; 434.91; 435.x-436.x;	I60.x-I64.x; G45.0-G45.3; G45.8-G45.9	NA
Erythrocytosis	NA	NA	NA	For male:<br>1. haemoglobin level higher than 16.5 g/dL;<br>or 2. haematocrit > 49%<br><br>For female:<br>1. haemoglobin level higher than 16.0 g/dL;<br>or 2. haematocrit > 48%

ICPC-2 = International Classification of Primary Care, Version 2; ICD-9-CM = International Statistical Classification of Diseases and Related Health Problems, 9th Revision, Clinical Modification; ICD-10-CM = International Statistical Classification of Diseases and Related Health Problems, 10th Revision, Clinical Modification; NA = not applicable

Table S2. Incidence rate ratios of event outcomes in different exposure periods of metformin and SGLT2i use

Events	Exposure periods	Number of events	Person-year	Mean (SD) observation period, years	Mean (SD) HbA1c, %	IRR	95% CI	P-value	IRR	95% CI	P-value
Cardiovascular diseases	Metformin only	1,625	9739.8	3.78 (1.46)	8.14 (1.21)		1 (Reference)		2.30	(1.95,2.71)	<0.001*
	Pre-SGLT2i	611	698.9	0.27 (0.08)	8.53 (1.63)	4.10	(3.65,4.60)	<0.001*	9.42	(8.10,10.95)	<0.001*
	Metformin & SGLT2i	310	3136.0	1.22 (1.13)	7.93 (1.28)	0.43	(0.37,0.51)	<0.001*		1 (Reference)	
	SGLT2i only	29	311.3	0.12 (0.43)	8.16 (1.65)	0.31	(0.20,0.48)	<0.001*	0.72	(0.47,1.10)	0.127
Coronary heart disease	Metformin only	1259	7726.9	3.75 (1.45)	8.09 (1.19)		1 (Reference)		2.26	(1.87,2.72)	<0.001*
	Pre-SGLT2i	515	561.9	0.27 (0.08)	8.52 (1.64)	4.41	(3.87,5.02)	<0.001*	9.95	(8.43,1174)	<0.001*
	Metformin & SGLT2i	258	2567.3	1.25 (1.12)	7.88 (1.25)	0.44	(0.37,0.53)	<0.001*		1 (Reference)	
	SGLT2i only	29	267.1	0.13 (0.45)	8.08 (1.57)	0.38	(0.25,0.59)	<0.001*	0.87	(0.56,1.34)	0.524
Hospitalisation for heart failure	Metformin only	472	3009.8	3.66 (1.53)	8.14 (1.38)		1 (Reference)		3.41	(2.51,4.62)	<0.001*
	Pre-SGLT2i	250	226.8	0.28 (0.08)	8.43 (1.74)	3.79	(3.12,4.60)	<0.001*	12.90	(9.78,17.01)	<0.001*
	Metformin & SGLT2i	85	801.5	0.98 (1.06)	8.08 (1.36)	0.29	(0.22,0.40)	<0.001*		1 (Reference)	
	SGLT2i only	15	162.0	0.20 (0.52)	8.34 (1.80)	0.17	(0.09,0.31)	<0.001*	0.57	(0.31,1.06)	0.076
Stroke	Metformin only	461	2718.5	3.73 (1.46)	8.37 (1.30)		1 (Reference)		1.04	(0.80,1.35)	0.763
	Pre-SGLT2i	103	200.3	0.28 (0.08)	8.68 (1.62)	2.66	(2.08,3.40)	<0.001*	2.77	(2.10,3.66)	<0.001*
	Metformin & SGLT2i	150	896.1	1.23 (1.17)	8.18 (1.42)	0.96	(0.74,1.25)	0.763		1 (Reference)	

	SGLT2i only	14	74.7	0.10 (0.38)	8.36 (1.62)	0.70	(0.36,1.36)	0.294	0.73	(0.38,1.42)	0.355
Erythrocytosis	Metformin only	571	5367.1	3.16 (1.50)	8.53 (1.29)		1 (Reference)		0.23	(0.20,0.27)	<0.001*
	Pre-SGLT2i	53	473.8	0.28 (0.09)	8.91 (1.57)	1.13	(0.84,1.51)	0.418	0.26	(0.20,0.35)	<0.001*
	Metformin & SGLT2i	990	3353.5	1.97 (1.44)	8.02 (1.23)	4.30	(3.65,5.06)	<0.001*		1 (Reference)	
	SGLT2i only	86	250.1	0.15 (0.54)	8.27 (1.52)	3.88	(2.69,5.61)	<0.001*	0.90	(0.63,1.28)	0.567

SGLT2i = sodium-glucose cotransporter-2 inhibitors; HbA1c = glycated haemoglobin; IRR = incidence rate ratio; SD = standard deviation; CI = confidence interval

The conditional Poisson regression models were adjusted by time-varying patients' age and the use of sulfonylureas, thiazolidinedione, dipeptidyl peptidase-4 inhibitors, glucagon-like peptide-1 receptor agonists, and insulin.

Table S3. Incidence rate ratios of cardiovascular diseases, coronary heart disease, hospitalisation for heart failure, stroke, and erythrocytosis in different exposure periods of metformin and dapagliflozin use

Events	Exposure periods	Number of events	Person-year	Mean (SD) observation period, years	Mean (SD) HbA1c, %	IRR	95% CI	P-value
Cardiovascular diseases	Metformin only	487	2934.4	3.86 (1.55)	8.16 (1.22)		1 (Reference)	
	Pre-dapagliflozin	158	206.9	0.27 (0.08)	8.52 (1.65)	3.79	(3.08,4.68)	<0.001*
	Metformin & dapagliflozin	107	896.8	1.18 (1.19)	8.04 (1.41)	0.57	(0.43,0.75)	<0.001*
	Dapagliflozin only	9	99.4	0.13 (0.49)	8.39 (1.84)	0.31	(0.14,0.68)	0.003*
Coronary heart disease	Metformin only	353	2199.8	3.87 (1.55)	8.05 (1.18)		1 (Reference)	
	Pre-dapagliflozin	128	154.7	0.27 (0.08)	8.51 (1.69)	3.99	(3.13,5.07)	<0.001*
	Metformin & dapagliflozin	77	660.8	1.16 (1.17)	7.97 (1.37)	0.53	(0.38,0.73)	<0.001*
	Dapagliflozin only	10	79.7	0.14 (0.51)	8.13 (1.65)	0.42	(0.19,0.90)	0.026*
Hospitalisation for heart failure	Metformin only	153	945.3	3.92 (1.56)	8.05 (1.31)		1 (Reference)	
	Pre-dapagliflozin	63	64.5	0.27 (0.07)	8.12 (1.67)	3.37	(2.34,4.83)	<0.001*
	Metformin & dapagliflozin	18	192.1	0.80 (1.05)	8.15 (1.55)	0.28	(0.15,0.51)	<0.001*
	Dapagliflozin only	7	53.6	0.22 (0.63)	8.77 (2.21)	0.25	(0.10,0.66)	0.005*
Stroke	Metformin only	164	933.5	3.66 (1.59)	8.41 (1.30)		1 (Reference)	
	Pre-dapagliflozin	35	70.0	0.27 (0.08)	8.72 (1.65)	2.51	(1.67,3.79)	<0.001*
	Metformin & dapagliflozin	51	321.7	1.26 (1.32)	8.32 (1.54)	0.89	(0.58,1.38)	0.603
	Dapagliflozin only	5	31.0	0.12 (0.44)	8.42 (1.53)	0.59	(0.20,1.71)	0.330
Erythrocytosis	Metformin only	233	2059.9	3.15 (1.64)	8.58 (1.29)		1 (Reference)	
	Pre-dapagliflozin	20	180.6	0.28 (0.08)	8.88 (1.53)	1.02	(0.64,1.63)	0.931
	Metformin & dapagliflozin	369	1328.6	2.03 (1.61)	8.09 (1.28)	3.33	(2.64,4.19)	<0.001*
	Dapagliflozin only	31	96.8	0.15 (0.58)	8.28 (1.75)	3.23	(1.77,5.90)	<0.001*

HbA1c = glycated haemoglobin; IRR = incidence rate ratio; SD = standard deviation; CI = confidence interval

The conditional Poisson regression models were adjusted by time-varying patients' age and the use of sulfonylureas, thiazolidinedione, dipeptidyl peptidase-4 inhibitors, glucagon-like peptide-1 receptor agonists, and insulin.

Table S4. Incidence rate ratios of cardiovascular diseases, coronary heart disease, hospitalisation for heart failure, stroke, and erythrocytosis in different exposure periods of metformin and empagliflozin use

Events	Exposure periods	Number of events	Person-year	Mean (SD) observation period, years	Mean (SD) HbA1c, %	IRR	95% CI	P-value
Cardiovascular diseases	Metformin only	1227	7302.9	3.83 (1.40)	8.16 (1.21)		1 (Reference)	
	Pre-empagliflozin	459	506.5	0.27 (0.06)	8.55 (1.63)	4.09	(3.57,4.69)	<0.001*
	Metformin & empagliflozin	201	2239.8	1.17 (1.05)	7.92 (1.27)	0.37	(0.31,0.46)	<0.001*
	Empagliflozin only	20	212.4	0.11 (0.39)	8.08 (1.56)	0.30	(0.18,0.49)	<0.001*
Coronary heart disease	Metformin only	974	5911.3	3.78 (1.39)	8.13 (1.20)		1 (Reference)	
	Pre-empagliflozin	391	418.3	0.27 (0.07)	8.55 (1.64)	4.43	(3.81,5.15)	<0.001*
	Metformin & empagliflozin	180	1905.8	1.22 (1.05)	7.89 (1.25)	0.41	(0.33,0.51)	<0.001*
	Empagliflozin only	19	187.8	0.12 (0.41)	8.09 (1.58)	0.36	(0.21,0.61)	<0.001*
Hospitalisation for heart failure	Metformin only	346	2225.5	3.64 (1.48)	8.20 (1.42)		1 (Reference)	
	Pre-empagliflozin	191	167.0	0.27 (0.08)	8.56 (1.79)	4.00	(3.19,5.00)	<0.001*
	Metformin & empagliflozin	67	609.8	1.00 (0.99)	8.10 (1.35)	0.31	(0.22,0.43)	<0.001*
	Empagliflozin only	8	105.7	0.17 (0.45)	8.19 (1.61)	0.14	(0.06,0.31)	<0.001*
Stroke	Metformin only	320	1909.8	3.83 (1.37)	8.35 (1.30)		1 (Reference)	
	Pre-empagliflozin	71	134.5	0.27 (0.07)	8.66 (1.62)	2.71	(2.01,3.67)	<0.001*
	Metformin & empagliflozin	98	575.4	1.16 (1.06)	8.12 (1.37)	0.96	(0.70,1.33)	0.807
	Empagliflozin only	9	43.8	0.09 (0.34)	8.33 (1.65)	0.72	(0.31,1.64)	0.431
Erythrocytosis	Metformin only	419	3790.8	3.35 (1.38)	8.51 (1.28)		1 (Reference)	
	Pre-empagliflozin	39	308.5	0.27 (0.08)	8.91 (1.61)	1.24	(0.87,1.75)	0.233
	Metformin & empagliflozin	620	2021.8	1.78 (1.25)	8.02 (1.23)	4.37	(3.55,5.38)	<0.001*
	Empagliflozin only	55	148.8	0.13 (0.48)	8.27 (1.38)	3.95	(2.50,6.24)	<0.001*

HbA1c = glycated haemoglobin; IRR = incidence rate ratio; SD = standard deviation; CI = confidence interval

The conditional Poisson regression models were adjusted by time-varying patients' age and the use of sulfonylureas, thiazolidinedione, dipeptidyl peptidase-4 inhibitors, glucagon-like peptide-1 receptor agonists, and insulin.

Table S5. Incidence rate ratios of cardiovascular diseases, coronary heart disease, hospitalisation for heart failure, stroke, and erythrocytosis in different exposure periods of metformin and SGLT2i use for patients who achieved targeted glycaemic control (HbA1c<7%) when initiated SGLT2i

Events	Exposure periods	Number of events	Person-year	Mean (SD) observation period, years	Mean (SD) HbA1c, %	IRR	95% CI	P-value	IRR	95% CI	P-value
Cardiovascular diseases	Metformin only	187	1377.0	3.99 (1.52)	7.23 (0.85)		1 (Reference)		4.26	(2.40,7.55)	<0.001*
	Pre-SGLT2i	136	92.3	0.27 (0.07)	6.68 (0.61)	6.91	(5.11,9.34)	<0.001*	29.41	(17.63,49.05)	<0.001*
	Metformin & SGLT2i	20	319.8	0.93 (1.00)	6.73 (0.64)	0.23	(0.13,0.42)	<0.001*		1 (Reference)	
	SGLT2i only	2	30.9	0.09 (0.27)	7.32 (2.08)	0.18	(0.04,0.81)	0.026*	0.78	(0.17,3.61)	0.755
Coronary heart disease	Metformin only	141	1118.8	3.93 (1.55)	7.24 (0.90)		1 (Reference)		3.95	(2.10,7.42)	<0.001*
	Pre-SGLT2i	126	76.8	0.27 (0.08)	6.71 (0.58)	8.34	(5.96,11.68)	<0.001*	32.92	(18.88,57.42)	<0.001*
	Metformin & SGLT2i	17	257.5	0.90 (0.95)	6.70 (0.56)	0.25	(0.13,0.48)	<0.001*		1 (Reference)	
	SGLT2i only	1	28.9	0.10 (0.29)	7.04 (1.12)	0.09	(0.01,0.69)	0.021*	0.34	(0.04,2.76)	0.311
Hospitalisation for heart failure	Metformin only	77	638.9	3.83 (1.61)	7.25 (1.04)		1 (Reference)		4.20	(1.81,9.77)	0.001*
	Pre-SGLT2i	78	45.3	0.27 (0.07)	6.57 (0.59)	7.64	(5.07,11.50)	<0.001*	32.09	(15.05,68.46)	<0.001*
	Metformin & SGLT2i	9	130.8	0.78 (0.97)	6.81 (0.77)	0.24	(0.10,0.55)	0.001*		1 (Reference)	
	SGLT2i only	3	26.8	0.16 (0.41)	7.48 (2.29)	0.33	(0.09,1.24)	0.102	1.40	(0.34,5.80)	0.646
Stroke	Metformin only	46	299.3	4.10 (1.49)	7.32 (0.98)		1 (Reference)		1.85	(0.68,5.05)	0.231

	Pre-SGLT2i	17	19.5	0.27 (0.06)	6.59 (0.85)	3.38	(1.68,6.81)	0.001*	6.25	(2.39,16.32)	<0.001*
	Metformin & SGLT2i	8	50.7	0.69 (0.92)	6.76 (0.89)	0.54	(0.20,1.48)	0.231	1 (Reference)		
	SGLT2i only	2	6.2	0.08 (0.18)	6.86 (1.59)	0.43	(0.08,2.41)	0.336	0.79	(0.13,4.84)	0.801
Erythrocytosis	Metformin only	43	411.9	3.19 (1.63)	7.29 (0.96)	1 (Reference)			0.22	(0.12,0.42)	<0.001*
	Pre-SGLT2i	12	35.0	0.27 (0.07)	6.68 (0.63)	3.43	(1.67,7.06)	0.001*	0.77	(0.39,1.53)	0.459
	Metformin & SGLT2i	66	219.9	1.70 (1.44)	6.84 (0.72)	4.45	(2.41,8.21)	<0.001*	1 (Reference)		
	SGLT2i only	8	27.6	0.21 (0.72)	7.61 (2.97)	2.92	(0.85,10.00)	0.087	0.66	(0.21,2.09)	0.477

SGLT2i = sodium-glucose cotransporter-2 inhibitors; HbA1c = glycated haemoglobin; IRR = incidence rate ratio; SD = standard deviation; CI = confidence interval

The conditional Poisson regression models were adjusted by time-varying patients' age and the use of sulfonylureas, thiazolidinedione, dipeptidyl peptidase-4 inhibitors, glucagon-like peptide-1 receptor agonists, and insulin.

Table S6. Incidence rate ratios of event outcomes in different risk periods by SGLT2i exposure and erythrocytosis

Events	Risk periods	IRR	95% CI	P	IRR	95% CI	P
Cardiovascular diseases	SGLT2i use with erythrocytosis	0.18	(0.11,0.28)	<0.001*	0.68	(0.43,1.07)	0.093
	SGLT2i use without erythrocytosis	0.26	(0.23,0.30)	<0.001*		1 (Reference)	
	Non-SGLT2i use with erythrocytosis	1.04	(0.72,1.50)	0.824			
	Non-SGLT2i use without erythrocytosis		1 (Reference)				
Coronary heart disease	SGLT2i use with erythrocytosis	0.22	(0.14,0.34)	<0.001*	0.92	(0.59,1.45)	0.724
	SGLT2i use without erythrocytosis	0.24	(0.20,0.28)	<0.001*		1 (Reference)	
	Non-SGLT2i use with erythrocytosis	0.89	(0.59,1.34)	0.575			
	Non-SGLT2i use without erythrocytosis		1 (Reference)				
Hospitalisation for heart failure	SGLT2i use with erythrocytosis	0.06	(0.02,0.14)	<0.001*	0.38	(0.14,0.99)	0.049*
	SGLT2i use without erythrocytosis	0.15	(0.11,0.19)	<0.001*		1 (Reference)	
	Non-SGLT2i use with erythrocytosis	0.79	(0.42,1.48)	0.459			
	Non-SGLT2i use without erythrocytosis		1 (Reference)				
Stroke	SGLT2i use with erythrocytosis	0.57	(0.26,1.24)	0.155	0.66	(0.30,1.42)	0.285
	SGLT2i use without erythrocytosis	0.87	(0.69,1.10)	0.243		1 (Reference)	
	Non-SGLT2i use with erythrocytosis	1.07	(0.54,2.14)	0.849			
	Non-SGLT2i use without erythrocytosis		1 (Reference)				

SGLT2i = sodium-glucose cotransporter-2 inhibitors; IRR = incidence rate ratio; CI = confidence interval

The conditional Poisson regression models were adjusted by time-varying patients' age, smoking status, use of diuretics, status of chronic obstructive pulmonary disease, and the use of sulfonylureas, thiazolidinedione, dipeptidyl peptidase-4 inhibitors, glucagon-like peptide-1 receptor agonists, and insulin.

Table S7. Incidence rate ratios of cardiovascular diseases, coronary heart disease, hospitalisation for heart failure, and stroke in different risk periods by any exposure to SGLT2i and the development of erythrocytosis, with sex-specific cut-off for haemoglobin

Events	Exposure periods	IRR	95% CI	P-value	IRR	95% CI	P-value
Cardiovascular diseases	SGLT2i use with erythrocytosis with haemoglobin >17.5 g/dL (for male) or >17.0 g/dL (for female)	0.34	(0.08,1.53)	0.160	1.31	(0.30,5.84)	0.721
	SGLT2i use with erythrocytosis with haemoglobin >16.5 - ≤17.5 g/dL (for male) or >16.0 - ≤17.0 g/dL (for female)	0.17	(0.11,0.27)	<0.001*	0.65	(0.41,1.03)	0.068
	SGLT2i use without erythrocytosis	0.26	(0.23,0.30)	<0.001*		1 (Reference)	
	Non-SGLT2i use with erythrocytosis	1.04	(0.72,1.50)	0.840			
	Non-SGLT2i use without erythrocytosis		1 (Reference)				
Coronary heart disease	SGLT2i use with erythrocytosis with haemoglobin >17.5 g/dL (for male) or >17.0 g/dL (for female)	0.20	(0.03,1.54)	0.122	0.83	(0.11,6.48)	0.860
	SGLT2i use with erythrocytosis with haemoglobin >16.5 - ≤17.5 g/dL (for male) or >16.0 - ≤17.0 g/dL (for female)	0.22	(0.14,0.34)	<0.001*	0.91	(0.58,1.44)	0.695
	SGLT2i use without erythrocytosis	0.24	(0.20,0.28)	<0.001*		1 (Reference)	
	Non-SGLT2i use with erythrocytosis	0.89	(0.59,1.34)	0.570			
	Non-SGLT2i use without erythrocytosis		1 (Reference)				
Hospitalisation for heart failure	SGLT2i use with erythrocytosis with haemoglobin >17.5 g/dL (for male) or >17.0 g/dL (for female)	0.19	(0.02,1.82)	0.151	1.33	(0.14,12.57)	0.805
	SGLT2i use with erythrocytosis with haemoglobin >16.5 - ≤17.5 g/dL (for male) or >16.0 - ≤17.0 g/dL (for female)	0.05	(0.02,0.13)	<0.001*	0.33	(0.11,0.93)	0.036*
	SGLT2i use without erythrocytosis	0.15	(0.11,0.19)	<0.001*		1 (Reference)	
	Non-SGLT2i use with erythrocytosis	0.79	(0.42,1.49)	0.469			
	Non-SGLT2i use without erythrocytosis		1 (Reference)				
Stroke [†]	SGLT2i use with erythrocytosis with haemoglobin >17.5 g/dL (for male) or >17.0 g/dL (for female)	NA	NA	NA	NA	NA	NA
	SGLT2i use with erythrocytosis with haemoglobin >16.5 - ≤17.5 g/dL (for male) or >16.0 - ≤17.0 g/dL (for female)	0.59	(0.27,1.27)	0.178	0.68	(0.31,1.46)	0.320
	SGLT2i use without erythrocytosis	0.87	(0.69,1.10)	0.242		1 (Reference)	
	Non-SGLT2i use with erythrocytosis	1.08	(0.54,2.16)	0.826	1.24	(0.62,2.51)	0.543
	Non-SGLT2i use without erythrocytosis		1 (Reference)		1.15	(0.91,1.46)	0.242

SGLT2i = sodium-glucose cotransporter-2 inhibitors; IRR = incidence rate ratio; CI = confidence interval; NA = not applicable

The conditional Poisson regression models were adjusted by time-varying patients' age, smoking status, use of diuretics, status of chronic obstructive pulmonary disease, and the use of sulfonylureas, thiazolidinedione, dipeptidyl peptidase-4 inhibitors, glucagon-like peptide-1 receptor agonists, and insulin.

† The number of stroke events were limited in the group of SGLT2i use erythrocytosis with haemoglobin > 17.5 g/dL (for male) or > 17.0 g/dL (for female), hence the incidence rate ratios were not presented.
